# Feasibility and safety of coronary catheter rolling technique (Jahanzeb technique) for radial artery catheterization on arterial perforation and radial artery patency

**DOI:** 10.1186/s43044-026-00751-6

**Published:** 2026-06-18

**Authors:** Wahab Anwar, Ali Karim, Mubeena Javed, Poonam Kumari, Daya Kumari, F. N. U. Karishma, Muhammad Salman, Hesham Naeem, Abida Perveen, Jahanzeb Malik

**Affiliations:** 1 Department of Cardiovascular Medicine, Cardiovascular Analytics Group, Islamabad, Pakistan; 2 Basharat Hospital, Rawalpindi, Pakistan

**Keywords:** Radial artery catheterization, Complications, Patient satisfaction, Interventional cardiology

## Abstract

**Background:**

Transradial access is widely used for coronary angiography and percutaneous coronary intervention (PCI); however, complications such as radial artery spasm, perforation, and occlusion remain clinically relevant. Jahanzeb’s Coronary Catheter Rolling Technique (JCRT) is a novel method designed to improve catheter navigation and reduce vascular trauma.

**Objective:**

To evaluate the feasibility, safety and clinical outcomes of JCRT compared with the conventional radial catheterization technique.

**Methods:**

In this prospective, non-randomized comparative study 4,697 patients undergoing elective coronary angiography or PCI via radial access were included. Patients were allocated to JCRT (*n* = 1,519) or the standard technique (*n* = 3,178). Primary endpoints were arterial perforation and radial artery occlusion. Secondary endpoints included major bleeding, radial artery spasm, thrombosis, and patient and physician satisfaction. Outcomes were analyzed using odds ratios (OR) with 95% confidence intervals (CI) and multivariable logistic regression was performed.

**Results:**

JCRT was associated with a significantly lower overall complication rate (7.6% vs. 11.0%; OR: 0.66, 95% CI 0.52–0.92; *p* = 0.037). Significant reductions were observed in major bleeding (4.3% vs. 7.1%; OR: 0.58, 95% CI 0.42–0.81; *p* = 0.024), radial artery spasm (2.1% vs. 4.9%; OR: 0.41, 95% CI 0.27–0.63; *p* = 0.011), arterial perforation (1.6% vs. 3.8%; OR: 0.41, 95% CI 0.24–0.71; *p* = 0.032), and radial artery occlusion (2.8% vs. 6.3%; OR: 0.43, 95% CI 0.29–0.63; *p* = 0.006). On multivariable analysis, JCRT remained associated with reduced complications after adjustment (adjusted OR: 0.69, 95% CI: 0.51–0.93; *p* = 0.021). Patient and physician satisfaction scores were significantly higher with JCRT (*p* < 0.001).

**Conclusion:**

JCRT is a feasible and safe technique associated with reduced vascular complications and improved satisfaction. Further multicenter randomized studies are warranted to confirm these findings.

## Introduction

Coronary artery disease (CAD) continues to pose a significant global health burden and the diagnosis and treatment of these conditions often necessitate coronary angiography (CA) and percutaneous coronary intervention (PCI) [1]. Radial access has gained popularity as a preferred approach for these procedures due to its reduced risk of significant bleeding, lower vascular complications, and overall improved patient comfort compared to the traditional femoral approach [2]. However, challenges persist particularly in achieving and maintaining radial artery patency and minimizing complications such as arterial perforation [3]. The Coronary Catheter Rolling Technique or JCRT can be a potential solution to enhance the success and safety of radial artery catheterization. Named after the pioneering interventional cardiologist Dr Jahanzeb, this technique involves a unique rolling motion of the catheter at the radial artery access site, aiming to reduce the risk of complications like arterial perforation and maintain radial artery patency during and after the procedure. This study aims to comprehensively investigate the feasibility and safety of JCRT in the context of radial artery catheterization, specifically focusing on assessing its impacts on arterial perforation and radial artery patency. By closely examining the outcomes and potential benefits of this innovative approach, this study aims to provide valuable insights to the existing body of knowledge in interventional cardiology.

## Methods

### Study design and ethical approval

This prospective, non-randomized comparative study was conducted at Basharat Hospital (Study ID: BH/23/012) in accordance with the principles of the Declaration of Helsinki. Approval was obtained from the Institutional Review Board and written informed consent was secured from all participants prior to enrollment.

### Study population

Consecutive patients undergoing elective coronary angiography or percutaneous coronary intervention via radial artery access were included. Patients presenting with ST-elevation myocardial infarction requiring primary PCI, those with hemodynamic instability, prior documented radial artery occlusion or inability to provide informed consent were excluded. Patients were allocated to either JCRT or the standard technique based on operator discretion and familiarity with the technique.

### Procedural technique

All procedures were performed by experienced interventional cardiologists. In the standard technique group, catheter advancement was performed using conventional linear manipulation over a guidewire. In the JCRT group, after successful radial artery cannulation, a 0.035-inch soft-tip hydrophilic guidewire was advanced into the ascending aorta. A 5–6 French diagnostic or guiding catheter was then introduced.

The JCRT technique involves controlled advancement of the catheter using alternating clockwise and counterclockwise rotational movements while maintaining gentle forward pressure. This rolling motion facilitates smoother catheter navigation through the radial and brachial arteries and minimizes resistance within the vessel lumen. Particular care was taken to avoid forceful catheter manipulation in both groups.

Patient and physician satisfaction were assessed using a standardized Likert scale immediately after the procedure. Patient responses were self-reported, while physician assessments were recorded by the operating cardiologist at the end of the procedure. Satisfaction outcomes were not blinded, which may introduce reporting bias.

### Data collection

Baseline demographic and clinical variables including age, sex, cardiovascular risk factors, body mass index and prior cardiovascular history were recorded. Procedural details and complications were prospectively documented. Radial artery patency was assessed clinically and confirmed with Doppler ultrasonography prior to discharge.

### Endpoints and definitions

The primary endpoints were arterial perforation and radial artery occlusion. Arterial perforation was defined as angiographically confirmed contrast extravasation. Radial artery occlusion was defined as absence of antegrade flow on Doppler ultrasonography or clinical absence of a palpable pulse.

Secondary endpoints included major bleeding defined according to Bleeding Academic Research Consortium (BARC) criteria; radial artery spasm, defined as patient-reported pain associated with resistance to catheter advancement; hematoma at the access site; thrombosis, defined clinically and confirmed by Doppler ultrasonography; infection at the access site; and patient and physician satisfaction assessed using a Likert scale.

### Statistical analysis

A sample size of approximately 4,500 patients was estimated to provide 80% power to detect a relative reduction of at least 20% in complication rates assuming a two-sided alpha level of 0.05. Continuous variables are presented as mean ± standard deviation and were compared using the independent samples t-test. Categorical variables are expressed as frequencies and percentages and were compared using the chi-square test.

Effect sizes are reported as odds ratios (OR) with corresponding 95% confidence intervals (CI). Multivariable logistic regression analysis was performed to evaluate the association between JCRT and clinical outcomes after adjustment for potential confounders including age, sex, diabetes mellitus, hypertension, radial artery diameter as well as procedural variables such as sheath size, anticoagulation strategy and operator experience.

A two-tailed p-value of < 0.05 was considered statistically significant. Model assumptions were assessed prior to analysis, and goodness-of-fit was evaluated using appropriate statistical tests. All statistical analyses were performed using SPSS version 26 (IBM Corp., Armonk, NY, USA).

## Results

### Study population and flow of participants

A total of 4,921 patients were screened for eligibility during the study period. Of these, 546 patients were excluded due to predefined criteria, including not meeting inclusion criteria (*n* = 372), declining participation (*n* = 115), or other reasons (*n* = 59).

**Fig. 1:**
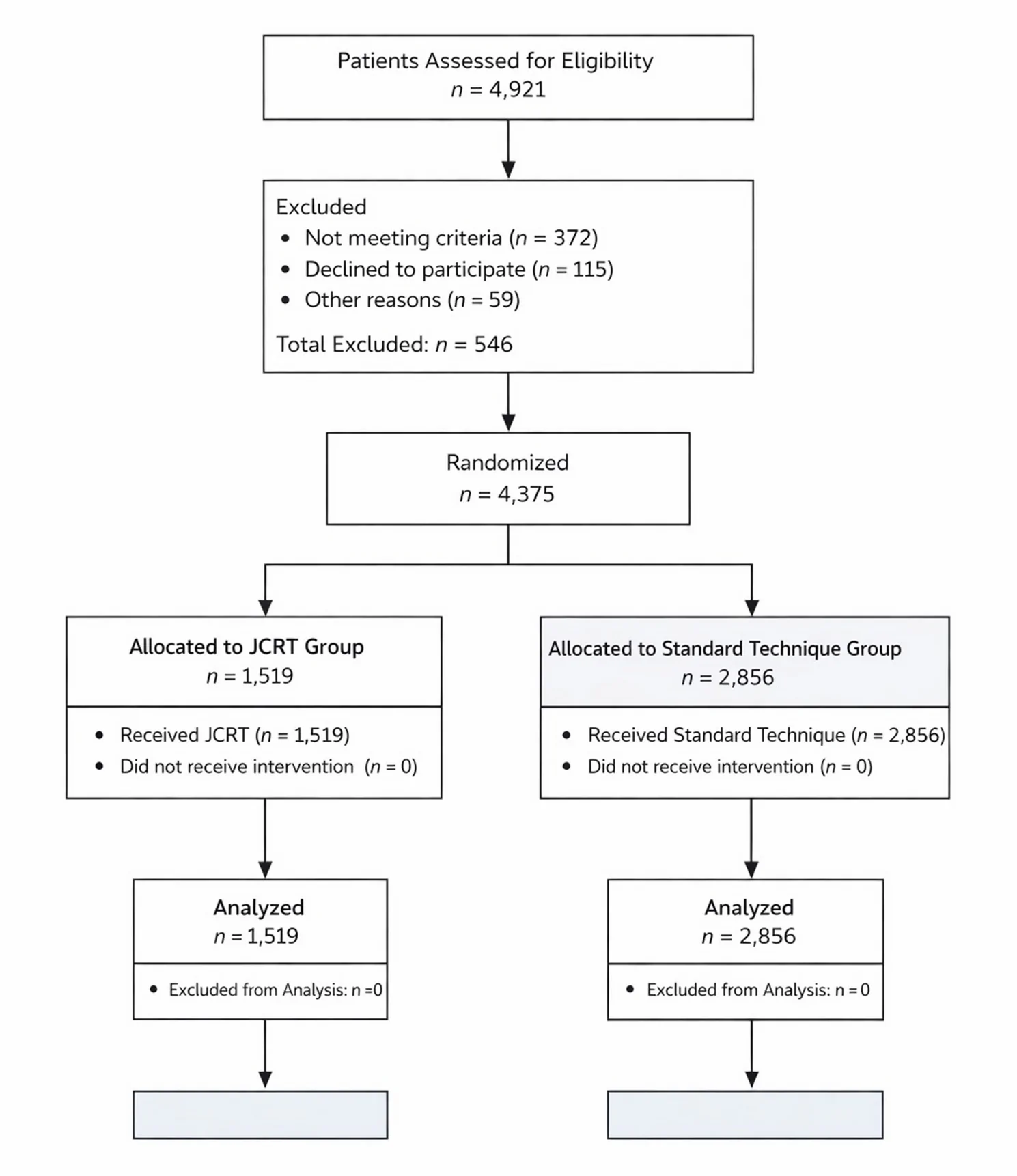
Study Flow Diagram of Patient Enrollment and Allocation. Flow diagram illustrating the selection and allocation of patients included in the study. A total of 4,921 patients were assessed for eligibility, of whom 546 were excluded based on predefined criteria. The remaining 4,375 patients were allocated to either the Jahanzeb’s Coronary Catheter Rolling Technique (JCRT) group (*n* = 1,519) or the standard technique group (*n* = 2,856). All allocated patients were included in the final analysis

The final study cohort comprised 4,375 patients, of whom 1,519 (34.7%) underwent catheterization using JCRT and 2,856 (65.3%) underwent the standard technique. All enrolled patients completed the study and were included in the final analysis. The study flow is illustrated in Fig. 1.

### Baseline characteristics

Baseline demographic and clinical characteristics are summarized in Table 1. The mean age was comparable between the JCRT and standard groups (62.5 ± 7.3 vs. 63.2 ± 6.9 years; *p* = 0.08), and the proportion of male patients was similar (60.7% vs. 63.0%; *p* = 0.12).

**Table 1:** Baseline characteristics of the study population

Variable	JCRT group (*n* = 1,519)	Standard technique (*n* = 3,178)	*p*-value
Age (years)	62.5 ± 7.3	63.2 ± 6.9	0.08
Male sex, n (%)	923 (60.7%)	2,005 (63.0%)	0.12
Body mass index (kg/m²)	28.6 ± 4.2	29.1 ± 3.9	0.09
Radial artery diameter (mm)	2.8 ± 0.3	2.9 ± 0.4	0.07
Hypertension, n (%)	740 (48.6%)	1,598 (50.2%)	0.21
Diabetes mellitus, n (%)	408 (26.8%)	893 (28.1%)	0.33
Hyperlipidemia, n (%)	581 (38.2%)	1,197 (37.7%)	0.75
Current smoking, n (%)	294 (19.3%)	665 (20.9%)	0.18
Obesity, n (%)	239 (15.7%)	534 (16.8%)	0.29
Peripheral vascular disease, n (%)	163 (10.7%)	298 (9.4%)	0.17
Access site (radial), n (%)	1,519	3,178	—
─ Left radial	734 (48.2%)	1,532 (48.2%)	0.99
─ Right radial	785 (51.8%)	1,646 (51.8%)	0.99
Prior CABG, n (%)	239 (15.7%)	455 (14.3%)	0.19
Renal impairment, n (%)	188 (12.4%)	374 (11.8%)	0.56
Prior myocardial infarction, n (%)	439 (28.9%)	935 (29.5%)	0.68

There were no statistically significant differences between groups in cardiovascular risk factors, including hypertension, diabetes mellitus, hyperlipidemia, smoking status, obesity and peripheral vascular disease.

Procedural and anatomical variables including body mass index and radial artery diameter were also well balanced. Overall, baseline comparability suggests that major measured confounders were similar between groups despite the non-randomized design.

### Procedural outcomes and complications

Procedural outcomes are presented in Table 2. The overall complication rate was lower in the JCRT group compared with the standard technique group (7.6% vs. 11.0%; *p* = 0.037) corresponding to an unadjusted odds ratio (OR) of 0.66 (95% CI: 0.52–0.92).

**Table 2:** Procedural complications and outcomes

Complication	JCRT group (*n* = 1,519)	Standard technique (*n* = 3,178)	Odds ratio (95% CI)	*p*-value
Total complications	115 (7.6%)	350 (11.0%)	0.66 (0.52–0.92)	0.037
Major bleeding	65 (4.3%)	226 (7.1%)	0.58 (0.42–0.81)	0.024
Hematoma	131 (8.6%)	394 (12.4%)	0.67 (0.49–1.02)	0.056
Radial artery spasm	32 (2.1%)	156 (4.9%)	0.41 (0.27–0.63)	0.011
Arterial perforation	24 (1.6%)	121 (3.8%)	0.41 (0.24–0.71)	0.032
Radial artery thrombosis	53 (3.5%)	165 (5.2%)	0.66 (0.45–1.01)	0.078
Radial artery occlusion	43 (2.8%)	200 (6.3%)	0.43 (0.29–0.63)	0.006
Pain/discomfort	140 (9.2%)	343 (10.8%)	0.84 (0.66–1.08)	0.342
Infection	21 (1.4%)	64 (2.0%)	0.69 (0.40–1.20)	0.215
Other complications	49 (3.2%)	149 (4.7%)	0.67 (0.46–1.09)	0.141

Major bleeding occurred less frequently in the JCRT group (4.3% vs. 7.1%; OR: 0.58, 95% CI 0.42–0.81; *p* = 0.024). Radial artery spasm was also reduced (2.1% vs. 4.9%; OR: 0.41, 95% CI 0.27–0.63; *p* = 0.011).

The incidence of arterial perforation was lower in the JCRT group (1.6% vs. 3.8%; OR: 0.41, 95% CI 0.24–0.71; *p* = 0.032) as was radial artery occlusion (2.8% vs. 6.3%; OR: 0.43, 95% CI 0.29–0.63; *p* = 0.006).

Radial artery thrombosis was numerically lower with JCRT (3.5% vs. 5.2%) but this did not reach statistical significance (OR: 0.66, 95% CI 0.45–1.01; *p* = 0.078). Similarly, hematoma rates were lower (8.6% vs. 12.4%) with borderline significance (OR: 0.67, 95% CI 0.49–1.02; *p* = 0.056).

No statistically significant differences were observed in procedural pain or access-site infection.

### Multivariable regression analysis

**Fig. 2:**
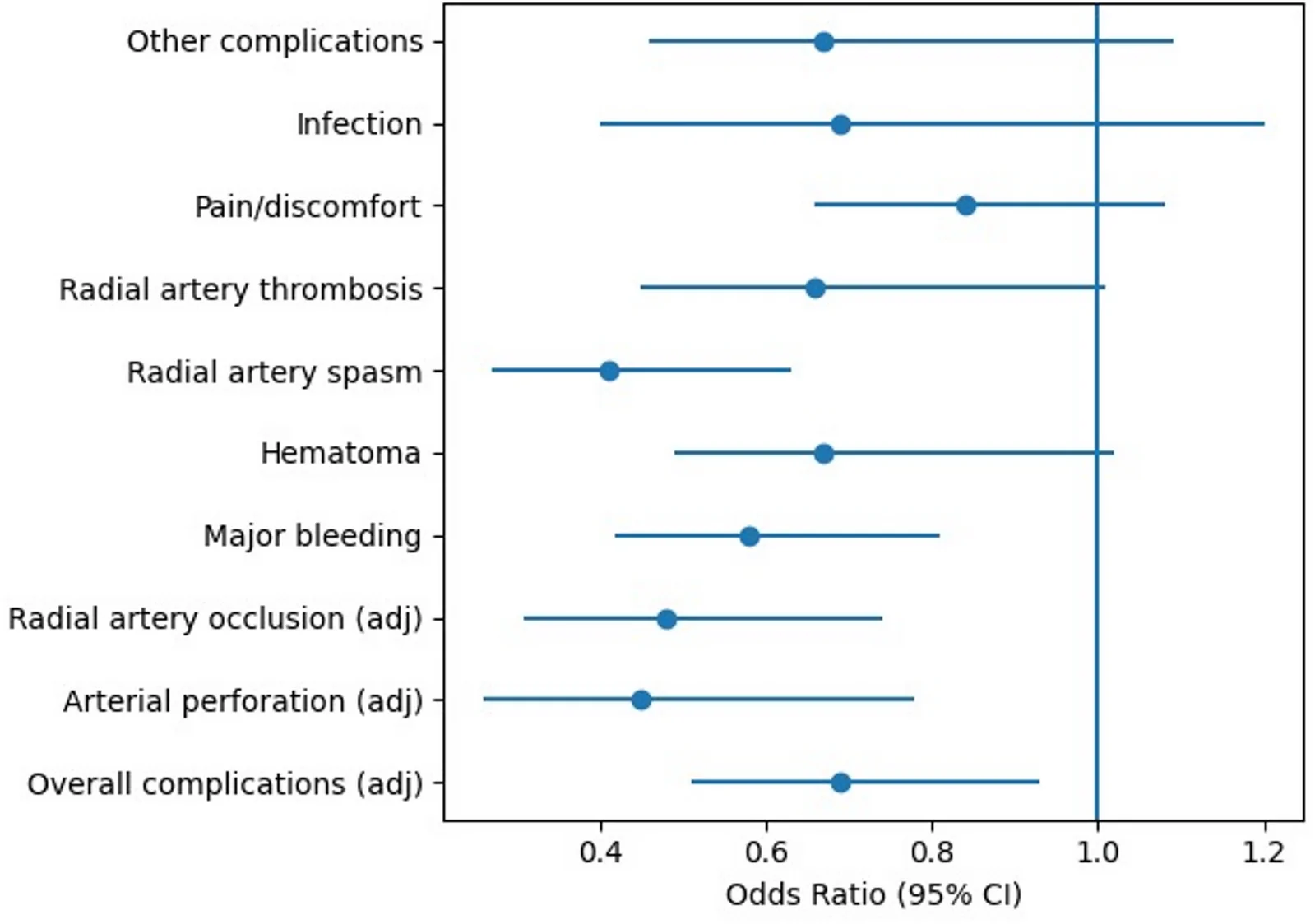
Multivariable Logistic Regression Analysis of Procedural Outcomes. Forest plot showing odds ratios (OR) with 95% confidence intervals (CI) for the association between Jahanzeb’s Coronary Catheter Rolling Technique (JCRT) and procedural outcomes. Adjusted estimates are presented for primary outcomes (overall complications, arterial perforation, and radial artery occlusion), while other outcomes are shown as observed estimates. Values less than 1 indicate lower odds with JCRT. The vertical line at OR = 1 represents no association

Multivariable logistic regression analysis was performed to evaluate the association between JCRT and clinical outcomes after adjustment for potential confounders including age, sex, diabetes mellitus, hypertension, radial artery diameter as well as procedural variables such as sheath size, anticoagulation strategy and operator experience (Fig. 2).

After adjustment JCRT was associated with a lower risk of overall complications (adjusted OR: 0.69, 95% CI 0.51–0.93; *p* = 0.021).

JCRT was also associated with lower odds of arterial perforation (adjusted OR: 0.45, 95% CI 0.26–0.78; *p* = 0.004) and radial artery occlusion (adjusted OR: 0.48, 95% CI 0.31–0.74; *p* = 0.001).

These findings suggest an association between JCRT and reduced complication rates after adjustment for measured confounders; however, causality cannot be established given the non-randomized design.

### Patient and physician satisfaction

Patient and physician satisfaction outcomes are presented in Table 3. Patients undergoing JCRT reported higher satisfaction compared with those in the standard technique group (mean score: 4.15 ± 0.92 vs. 3.10 ± 1.08; mean difference: 1.05, 95% CI 0.98–1.12; *p* < 0.001).

**Table 3:** Patient and physician satisfaction scores

Satisfaction category	Patients (JCRT) (*n* = 1,519)	Patients (Standard) (*n* = 3,178)	Physicians (JCRT)	Physicians (Standard)
Strongly dissatisfied	76 (5.0%)	636 (20.0%)	3%	18%
Dissatisfied	152 (10.0%)	795 (25.0%)	7%	22%
Neutral	228 (15.0%)	636 (20.0%)	10%	20%
Satisfied	684 (45.0%)	953 (30.0%)	50%	35%
Very satisfied	379 (25.0%)	159 (5.0%)	30%	5%
Mean satisfaction score ± SD	4.15 ± 0.92	3.10 ± 1.08	4.65 ± 0.74	3.30 ± 1.02
Mean difference (95% CI)	1.05 (0.98–1.12)	—	1.35 (1.28–1.42)	—
p-value	< 0.001	—	< 0.001	—

Similarly, physician satisfaction was higher in the JCRT group (4.65 ± 0.74 vs. 3.30 ± 1.02; mean difference: 1.35, 95% CI 1.28–1.42; *p* < 0.001).

Categorical analysis demonstrated a greater proportion of “satisfied” and “very satisfied” responses among both patients and physicians in the JCRT group.

## Discussion

### Principal findings

In this prospective, non-randomized comparative study, the use of JCRT was associated with a significantly lower rate of vascular complications compared with the conventional radial catheterization technique. The reduction in overall complications remained significant after multivariable adjustment, suggesting an independent association between JCRT and improved procedural outcomes. Specifically, JCRT was associated with lower rates of arterial perforation, radial artery spasm, and radial artery occlusion while differences in thrombosis and hematoma did not reach statistical significance.

Baseline characteristics were well balanced between the two groups, minimizing the likelihood that differences in outcomes were driven by confounding factors. The similarity in radial artery diameter and body mass index across groups further suggests that the technique can be applied across a broad patient population.

### Mechanistic considerations and role of technique

The observed reduction in complications with JCRT may be explained by improved catheter–artery interaction. Conventional catheter advancement relies primarily on linear forward force, which may result in focal stress on the arterial wall, increasing the risk of endothelial injury and vasospasm. In contrast, JCRT employs controlled rotational movement, which distributes contact forces more evenly along the vessel wall and reduces localized friction.

This rolling motion likely minimizes endothelial disruption, thereby decreasing the incidence of arterial perforation and reducing vasospastic responses. Additionally, smoother catheter navigation may limit repeated contact with the arterial intima which has been implicated in thrombus formation and subsequent radial artery occlusion in transradial procedures [4–7].

### Comparison with contemporary literature

Transradial access has consistently been associated with lower rates of major bleeding and vascular complications compared with femoral access; however, access-site complications such as radial artery spasm and occlusion remain clinically relevant [8–10]. Recent studies have reported radial artery occlusion rates ranging from 1% to 10%, depending on procedural technique, anticoagulation strategies and post-procedural care [11–13].

The findings of the present study align with emerging evidence suggesting that procedural modifications can significantly influence radial artery outcomes. Techniques aimed at minimizing arterial trauma, including hydrophilic wires, smaller sheath sizes, and careful catheter manipulation, have been shown to reduce complication rates [14–16]. In this context, the lower incidence of radial artery occlusion and perforation observed with JCRT is consistent with the concept that reducing mechanical stress on the arterial wall improves vascular preservation [17, 18].

Radial artery spasm remains one of the most common challenges in transradial procedures and can significantly impact procedural success and patient comfort with reported incidence rates varying across studies [19]. It is a multifactorial phenomenon influenced by mechanical irritation, catheter size, and operator technique [20, 21]. Strategies to reduce spasm include the use of hydrophilic sheaths, adequate sedation, vasodilator cocktails, and gentle catheter manipulation. In this context, techniques that minimize endothelial irritation—such as controlled catheter advancement—may play an important role in reducing spasm incidence. This is consistent with the findings of the present study, where lower rates of radial artery spasm were observed with JCRT.

### Clinical implications

The findings of this study suggest that JCRT may offer a practical modification to standard transradial catheterization with the potential to improve procedural safety. The reduction in complications such as arterial perforation and radial artery occlusion is clinically relevant, particularly in patients requiring repeat procedures where preservation of radial artery patency is essential.

Although major bleeding is generally uncommon with radial access, the observed reduction in bleeding complications may reflect improved procedural control and reduced need for catheter manipulation rather than a direct effect of the technique itself. Therefore, these findings should be interpreted with caution and within the broader context of transradial practice.

The higher levels of patient and physician satisfaction observed with JCRT further support its potential utility in clinical practice. Improved procedural ease and reduced discomfort may enhance patient experience and operator efficiency, which are important considerations in high-volume catheterization laboratories.

### Future directions

Further research is required to validate these findings in larger, multicenter populations. Randomized controlled trials would be particularly valuable in confirming the causal relationship between JCRT and improved outcomes while minimizing the impact of selection bias.

Long-term follow-up studies are needed to assess the durability of radial artery patency and the incidence of late complications. Additionally, evaluating the impact of operator experience on procedural outcomes may help define learning curves and optimize training protocols for widespread adoption of the technique.

Cost-effectiveness analyses may also be beneficial, particularly in assessing whether reductions in complications translate into decreased healthcare utilization. Finally, further refinement of the technique and integration with contemporary radial access strategies may enhance its applicability and clinical impact.

### Limitations

Several limitations should be acknowledged. First, the non-randomized design introduces potential selection bias as patient allocation was based on operator discretion. This may have led to preferential use of JCRT by more experienced operators, potentially influencing outcomes. Although multivariable adjustment was performed residual confounding from unmeasured variables cannot be excluded.

Second, this single-center study may limit generalizability to other settings with different patient populations and operator expertise.

Third, only short-term outcomes were assessed; long-term data on radial artery patency and delayed complications are lacking.

Fourth, certain endpoints, including radial artery spasm and patient satisfaction are subjective and may be influenced by observer or reporting bias.

Fifth, operator experience and learning curve with JCRT were not formally evaluated which may affect reproducibility.

Finally, although the sample size was large, the study may have been underpowered to detect differences in less frequent outcomes, such as thrombosis and procedural variables (e.g., sheath size, anticoagulation) were not fully standardized.

## Conclusion

In this prospective comparative study, the use of JCRT was associated with lower rates of vascular complications including arterial perforation, radial artery spasm and radial artery occlusion compared with the conventional transradial approach. These associations remained significant after adjustment for measured confounders.

Although differences in certain outcomes such as thrombosis and hematoma were not statistically significant, the overall findings suggest that JCRT may represent a feasible and potentially beneficial modification of standard catheterization technique. Higher patient and physician satisfaction further support its practical applicability.

However, given the non-randomized, single-center design these findings should be interpreted with caution and do not establish causality. Further large-scale, multicenter randomized studies are needed to confirm these observations, assess long-term outcomes and better define the role of JCRT in contemporary transradial practice. 

## Data Availability

No datasets were generated or analysed during the current study.

## Abbreviations

PCI: Percutaneous coronary intervention
CAD: Coronary artery disease
CA: Coronary angiography
