# Correction: Feasibility and safety of coronary catheter rolling technique (Jahanzeb technique) for radial artery catheterization on arterial perforation and radial artery patency

**DOI:** 10.1186/s43044-026-00789-6

**Published:** 2026-10-15

**Authors:** Wahab Anwar, Ali Karim, Mubeena Javed, Poonam Kumari, Daya Kumari, F. N. U. Karishma, Muhammad Salman, Hesham Naeem, Abida Perveen, Jahanzeb Malik

**Affiliations:** 1 Department of Cardiovascular Medicine, Cardiovascular Analytics Group, Islamabad, Pakistan; 2 Basharat Hospital, Rawalpindi, Pakistan

**Correction: Egypt Heart J 78, 47 (2026)**.

https://doi.org/10.1186/s43044-026-00751-6.

Following publication of the original article [1], the authors identified an error in the author name of Mubeena Javed.

The incorrect author name is: Mubeena Javaid.

The correct author name is: Mubeena Javed.

The author group has been updated above and the original article [1] has been corrected.
